# Os odontoideum in wolcott-rallison syndrome: a case series of 4 patients

**DOI:** 10.1186/s13023-016-0397-z

**Published:** 2016-02-10

**Authors:** R. P. Dias, C. R. Buchanan, N. Thomas, S. Lim, G. Solanki, SEJ Connor, T. G. Barrett, R. R. Kapoor

**Affiliations:** Department of Paediatric Endocrinology and Diabetes, Birmingham Children’s Hospital, Birmingham, B4 6NH UK; Department of Child Health, King’s College Hospital, London, SE5 9RS UK; Department of Neurosurgery, Kings College Hospital, London, SE5 9RS UK; Department of Paediatrics, St John’s Hospital, Chelmsford, Essex CM2 9BG USA; Department of Neurosurgery, Birmingham Children’s Hospital, Birmingham, B4 6NH UK; Department of Neuroradiology, Kings College Hospital, London, SE5 9RS UK; Centre for Rare Diseases and Personalized Medicine, Institute of Biomedical Research (West), School of Clinical and Experimental Medicine, University of Birmingham, Birmingham, B15 2TT UK

**Keywords:** Neonatal Diabetes, Os Odontoideum, Wolcott-Rallison Syndrome, Atlanto-axial instability

## Abstract

Wolcott-Rallison Syndrome is the commonest cause of neonatal diabetes in consanguineous families. It is associated with liver dysfunction, epiphyseal dysplasia, and developmental delay. It is caused by mutations in eukaryotic translation initiation factor 2-α kinase 3 (*EIF2AK3*).

We report 4 children with WRS and Os Odontoideum resulting in significant neurological compromise. This cervical spine abnormality has not previously been described in this syndrome. This additional evidence broadens the clinical spectrum of this syndrome and confirms the role of *EIF2AK3* in skeletal development. Furthermore, Os Odontoideum needs to be actively screened for in WRS patients to prevent neurological and respiratory compromise.

## Letter to the editor

Wolcott-Rallison Syndrome (WRS; OMIM 226980) is a rare autosomal recessive disorder characterised by infancy onset insulin-dependent diabetes and multiple epiphyseal dyspasia [1]. It is caused by mutations in the gene encoding eukaryotic translation initiation factor 2-α kinase 3 (*EIF2AK3*) which is located at Chromosome 2p12 [2]. EIF2AK3 acts as a stress sensor and, upon activation, phosphorylates EIF2alpha and regulates synthesis of unfolded proteins in the endoplasmic reticulum [3]. Other reported clinical features of WRS include recurrent episodes of severe (potentially fatal) hepatic dysfunction, renal impairment and developmental delay [4, 5]. The multiple epiphyseal dysplasia associated with WRS characteristically affects the long bones, pelvis and vertebrae. Osteoporosis has also been described in association with WRS in some patients [6].

We report four patients with WRS and Os odontoideum, a cervical spine abnormality not previously reported in associated with WRS. Os odontoideum is a condition where the odontoid peg is radiologically separated from the body of the second cervical vertebra although continuity with cartilagenous (unossified) tissues has not been clearly defined [7]. Two of the four patients had symptomatic atlanto-axial instability (AAI) requiring spinal fusion to relieve symptoms.

## Case reports

### Patient 1

This female patient, now aged 21 years, presented with antibody negative (Islet Cell / Glutamic Acid decarboxylase) diabetes mellitus aged 6 months. Her parents are White British and unrelated. At 3 years of age, she developed acute liver failure, encephalopathy and renal dysfunction during a minor febrile illness. She recovered completely except for residual cerebellar signs (dysarthria, ataxia). Progressive growth failure developed from 3–4 years of age prompting assessment of pituitary function which did not reveal any abnormality. Hand radiography revealed acro-osteolysis of distal phalanges 1,3 and 5 and the dysplastic carpal bones. Subsequent skeletal survey confirmed multiple epiphyseal dysplasia with hip subluxation and extensive calcification of soft tissues around the knee joints. WRS was confirmed on genetic testing which revealed compound heterozygosity for a frameshift mutation c.577delA and a missense mutation R632W (c.1966C > T) in exons 3 and 12 of the *E1F2AK3* gene, (previously reported [8].

**Fig. 1:**
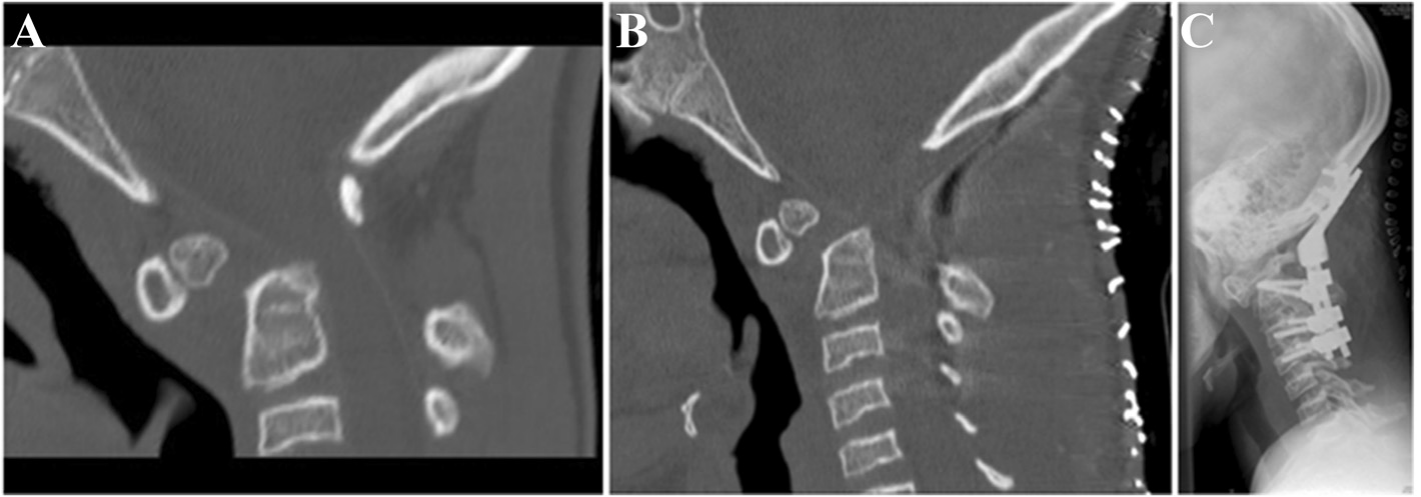
Radiological images from Case 1. **a**) Pre-operative sagittal midline CT (on bone windows) shows an os odontoideum widely separated from the body of C2 with a widening of the anterior atlanto-dental distance. There is a marked reduction in the sagittal bony canal dimensions between C2 body and the C1 posterior arch) **b**) Post-operative sagittal midline CT (on bone windows) demonstrates resection of the posterior arch of atlas and some reduction of the os odontoideum with a reduced distance between the os and the body of C2. **c**) Lateral plain radiograph demonstrates the occipito-cervical rod and screw fixation

Aged 11 years, worsening gait, lower limb pain, reduced mobility and evolution of upper motor neurone long tract signs led to radiological evaluation of cervical anatomy. Neuroimaging revealed an Os Odontoideum with spinal cord compression (Fig. 1a). Imaging of the neck showed normal position of the C1 anterior vertebral body and C2 main body with absence of ossification of the main body of the odontoid peg (Fig. 1a). The odontoid peg tip was normally sited in relation to the C1 vertebra but had become distracted from the main body of C2. The posterior body of C1 was normally positioned and readily visible in the sagittal view (Fig. 1a). The patient underwent successful occipito-cervical fusion to preserve the spinal canal and alleviate bone pressure on the spinal cord (fixation with pedicle screws into C2 and subaxial lateral mass screws and decompression with removal of the C1 lamina), (Fig. 1 b and c). The patient made an uneventful recovery from surgery with improvement of her myelopathic symptoms and signs.

### Patient 2

**Fig. 2:**
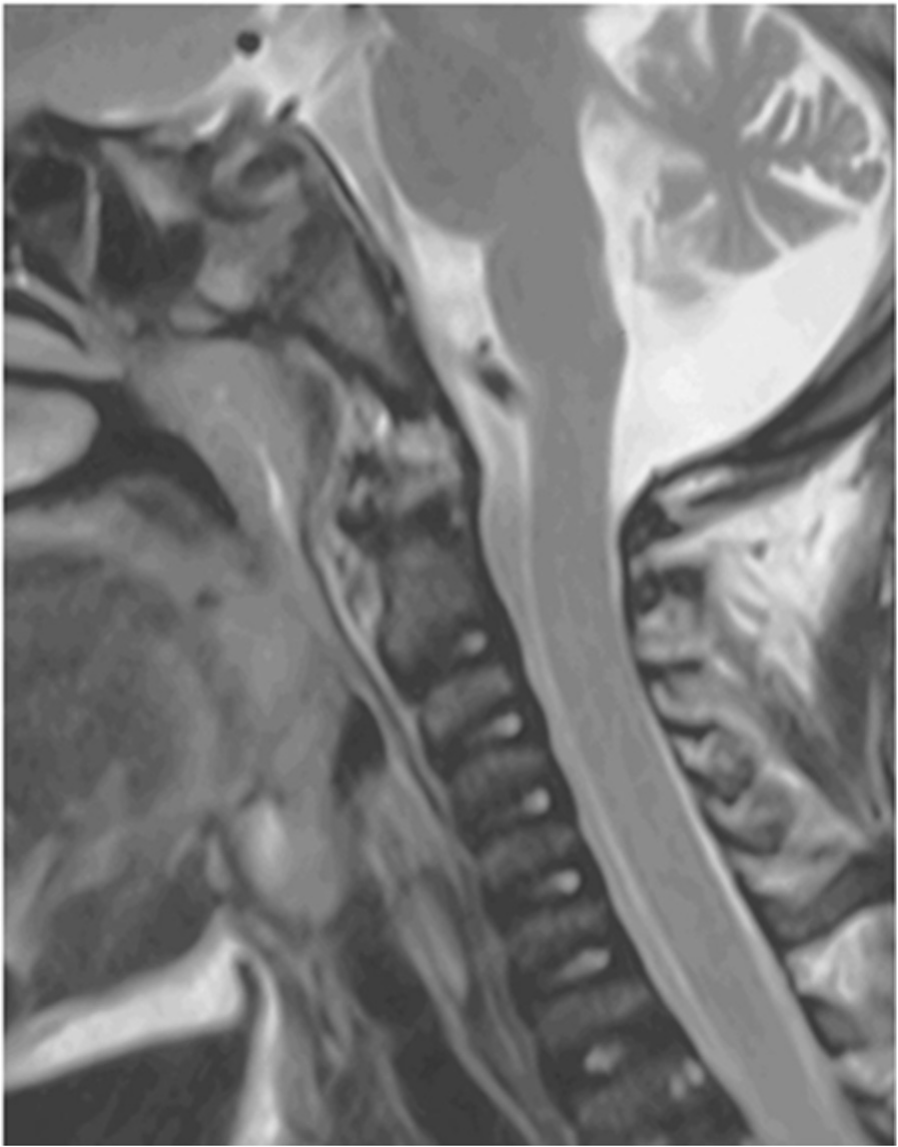
Radiological image from Case 2. Midline sagittal T2w image demonstrates lack of ossification of the dens

This male patient presented with diabetes mellitus and concurrent acute hepatic and renal dysfunction at 11 months of age. Detailed clinical and molecular genetic findings confirming the diagnosis of WRS is published elsewhere but he was found to have a homozygous frameshift mutation in exon 9 (c.1635_1638delGAAA) of *EIF2KA3* [8]. The diagnosis of Os odontoideum in case 1 prompted imaging in this patient at 9 years of age. The sagittal MRI view confirmed lack of ossification of the odontoid peg (Fig. 2). Imaging did not identify AAI and without clinically apparent neurological consequences of the odontoid abnormality and therefore, a conservative approach to management was adopted. The patient unfortunately died at the age of 12 years from acute liver failure.

### Patient 3

Case 3, aged 14.5 years at the most recent assessment has previously been reported in a case series when 4 years old [9]. He is the youngest of five siblings from consanguineous Pakistani parents. His oldest sister also has WRS and both carry the same homozygous missense mutation in *EIF2AK3* (c.1832G > A, R587Q) [2].

He presented aged 10 weeks with an upper respiratory tract infection, vomiting and hyperglycaemia. His diabetes remains poorly controlled with HbA1c persistently greater than 8 %. He was noted to have elevated liver enzymes and percutaneous liver biopsy showed progressive fibrosing intrahepatic cholestasis. Skeletal radiography revealed dysplastic femoral epiphyses.

**Fig. 3:**
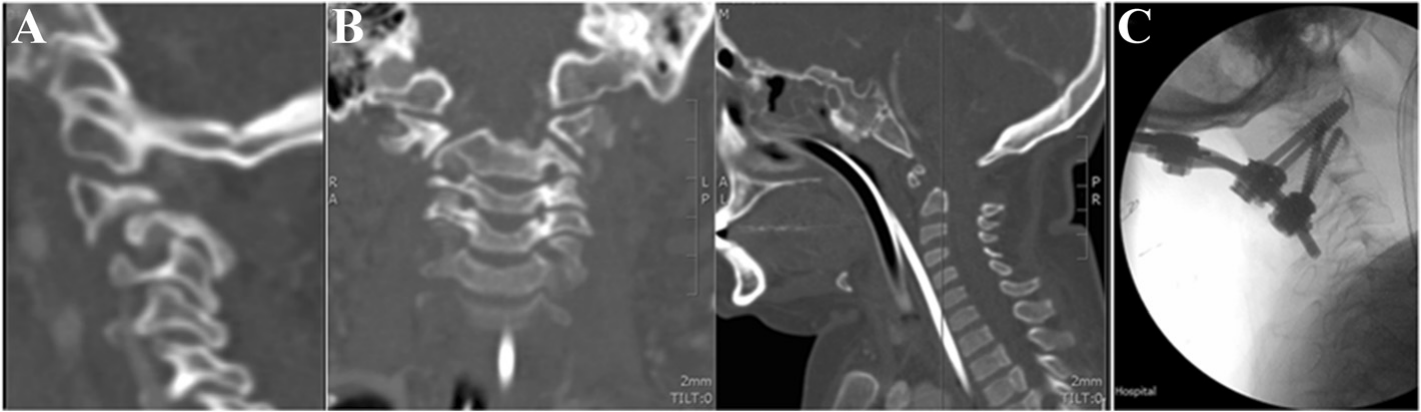
Radiological images from Case 3. **a** Pre-operative CT scan showing C1-2 subluxation with antero-inferior subluxation of C1 lateral mass on C2; (**b**) pre-operative MRI images showing coronal and sagittal views of C2 demonstrating congenital dens hypoplasia with central dip typical of embryological abnormality and absence of posterior arch of C1; (**c**) post-operative XR image showing occipital C2 fixation following C1-2 reduction (C1 now in line with C2)

At 3.8 years, he presented with encephalitis associated with significant loss of gross and fine motor skills, acute hepatitis and consistent EEG findings. Over the next 4 years his motor development improved, although remained delayed. He became wheelchair bound aged 7 years when imaging identified a 12 mm anterior translation of C1 on C2 flexion (Fig. 3a) He was initially managed conservatively with a cervical hard collar. MRI scan (Fig. 3b) confirmed AAI and over the next 3 years, neuro-surgical opinions repeatedly recommended surgical fixation. The family declined to follow this advice because of concerns regarding surgical risk despite progressive deterioration both on MRI and clinical assessment of upper and lower limb neurological signs, (reduced power and increased tone). Aged 11.9 years, he presented in cardio-respiratory arrest secondary to spinal cord injury. He was resuscitated successfully and underwent surgical C1-C2 posterior fixation (Fig. 3c). Following surgery, he was paraplegic and required long-term home ventilation. He died aged 15 years following an episode of acute liver failure.

### Patient 4

This male patient is now 4 years of age and the younger sibling of case 2. He had the same homozygous mutation (c.1635_1638delGAAA) of *EIF2KA3*. He presented aged 4 months of age with abnormal liver enzymes typical of WRS and diabetes. Due to his brother’s diagnosis he was tested for WRS and found to have the same mutation. His diabetes is controlled well on continuous subcutaneous insulin infusion. He has had recurrent episodes of acute liver dysfunction. Aged 3.5 years he developed right-sided focal seizures associated with left hemisphere focal epileptiform discharge on EEG and some evidence of cerebral atrophy in the left hemisphere. These are improving on anti-epileptic medication (ethosuximide, levetiracetam and clobazam). Cranioverterbral imaging showed os odontodium to be present, but with no instability on flexion-extension x-rays. Present management is conservative, as with his brother in view of the lack of AAI.

## Discussion

There are fewer than 60 published cases of WRS in the literature event though, it is the most common cause of neonatal diabetes in patients of consanguineous parents [4]. To date, there appears to be no clear genotype-phenotype correlation, even within a family as seen with patient 3 whose sibling has neither hepatic dysfunction nor evidence of AAI [5]. Early-onset diabetes, skeletal dysplasia and deranged liver enzymes are the cardinal features of WRS, and all the patients reported here demonstrate these defects. Additional reported features including renal impairment, neutropenia, hypothyroidism and developmental delay are much more variable [10]. Although atlanto-axial instability has previously been reported in this condition, radiological imaging was not shown and os odontoideum was not a reported feature in this single case [11]. Here we report 2 siblings in addition to 2 further unrelated cases, with os odontoideum an upper cervical spine anomaly not previously reported in WRS but of potentially high clinical significance. Os odontoideum is uncommon, characterised by the appearance of a corticated oval or round-shaped ossicle of variable size, representing the odontoid process but with failure to show ossified continuity with the body of the axis [12]. It can lead to AAI and spinal cord compression. Two of the four patients reported here had significant AAI causing neurological changes and required cervical spine fixation. As seen in case 3, the condition has potentially devastating consequences if AAI is left untreated. Although the siblings (Cases 2 and 4) show evidence of the condition occurring as a consistent phenotype with their particular gene mutation, this cannot be extrapolated given the small numbers involved and the lack of OO in the sibling of Case 3 (not presented here). We have a further 7 patients across the two centres with genetically confirmed WRS. 5 of these are younger than 5 years of age (age at which ossification is thought to complete) and hence although they have no evidence of neurological deficits or abnormalities on cervical spine X-rays, we cannot exclude later development of OO and/or AAI. A further case was only genetically confirmed post-mortem and so screening had not been undertaken. The final case is the older sister of Case 3 and has no evidence of OO or AAI on screening at present.

The aetiology of Os odontoideum has been attributed to congenital or traumatic mechanisms although current thinking is that the latter is more likely [12]. The presence of this rare anomaly in three unrelated patients with WRS suggests a congenital mechanism in our cases. EIF2AK3 is known to be induced by ER stress caused by malfolded proteins and leads reduction of translational initiation and repression of protein synthesis as well as G1 growth arrest with pleiotropic effects. Although the mechanism by which EIF2AK3 effects abnormal skeletal development and defective bone mineralisation, is as yet unknown, it has a clear role in these processes. This is not only demonstrated by patients with WRS but also in knockout mice who show a number of skeletal defects including deficient mineralization, osteoporosis, and abnormal compact bone development [13]. In addition, researchers have found an association with common variants in EIF2AK3 and reduced bone mineral density (although this is limited data, and only suggestive) [14]. Os odontoideum has also been described in association with other syndromes, with Downs syndrome being the commonest syndrome described previously [15, 16]. Hajdu-Cheney syndrome is also associated with acro-osteolysis of the phalanges and rarely cervical instability, which might suggest a common but as yet unidentified mechanism for the Os odontoideum [17]. However, it has also been suggested that the associated ligamentous laxity and defective ossification in these syndromes predisposes children with these underlying conditions to traumatic Os odontoideum [12]. Similarly, in WRS, it is possible that the underlying bony abnormalities (osteopenia and defective ossification) predispose the development of traumatic Os odontoideum. Multiple fractures frequently observed in WRS favour this hypothesis of a traumatic/acquired lesion in the background of congenital predisposition.

Os odontoideum may be asymptomatic or may cause symptoms due to spinal cord compression or vertebral artery compression [12] and symptoms may be transient [16]. The diagnosis of this anomaly is based on plain radiographs, assisted by computed tomography and magnetic resonance imaging techniques. If Os odontoideum is associated with AAI or neurological deficits, neurosurgical intervention is warranted [12]. Surgical fixation has the potential to prevent devastating consequences although equally carries its own significant risk which families and the medical team may decide is too great to carry out the intervention. We therefore recommend that assessment of children with WRS should include an appropriate and thorough regular neurological assessment, including imaging from diagnosis.
